# Scavenging reactive oxygen species using tempol in the acute phase of renal ischemia/reperfusion and its effects on kidney oxygenation and nitric oxide levels

**DOI:** 10.1186/s40635-015-0057-y

**Published:** 2015-07-04

**Authors:** Ugur Aksu, Bulent Ergin, Rick Bezemer, Asli Kandil, Dan M J Milstein, Cihan Demirci-Tansel, Can Ince

**Affiliations:** Department of Translational Physiology, Academic Medical Center, University of Amsterdam, Meibergdreef 9, 1105 AZ Amsterdam, The Netherlands; Department of Biology, Faculty of Science, Istanbul University, Vezneciler, Istanbul, 34459 Turkey

**Keywords:** Superoxide dismutase, Tempol, Tempo, Ischemia, Reperfusion, Renal, Microcirculation, Oxygenation, Nitric oxide

## Abstract

**Background:**

Renal ischemia/reperfusion (I/R) injury is commonly seen in kidney transplantation and affects the allograft survival rates. We aimed to test our hypothesis that scavenging reactive oxygen species (ROS) with tempol would protect renal oxygenation and nitric oxide (NO) levels in the acute phase of renal I/R.

**Methods:**

Rats were randomly divided: (1) no I/R, no tempol; (2) no I/R, but with tempol; (3) I/R without tempol; and (4) I/R with tempol. I/R was induced by 30-min clamping of the renal artery. Tempol (200 μmol/kg/h/i.v) was administered 15 min prior to I/R.

**Results:**

I/R without tempol led to a significant decrease in renal oxygen delivery and microvascular oxygenation. Tempol, however, protected renal oxygenation after I/R. At R90, the creatinine clearance rate was lower in the I/R-subjected group that did not receive tempol compared to that in the other groups. I/R injury without tempol treatment led to a significant increase in tissue malondialdehyde levels and a significant decrease in tissue NO levels. Tempol administration before I/R could prevent oxidative stress and altered tissue NO levels.

**Conclusions:**

This underscores that unbalance between oxygen, NO, and ROS forms an important component of the pathogenesis of I/R-induced AKI and should therefore be taken into account when designing a prevention/treatment strategy for renal I/R injury in transplantation.

## Background

Acute kidney injury (AKI) is a complex clinical complication and is associated with a high incidence of morbidity and mortality [1, 2]. One of the most common causes of AKI is renal ischemia/reperfusion (I/R) injury as it can occur in numerous scenarios such as during surgery and also as a result of shock (low perfusion), resuscitation (normal or even high perfusion), and renal transplantation [3–5]. Post-transplantation dysfunction is described as a delayed graft function, and the related pathophysiology of AKI is still incompletely understood, despite the identification of several mechanisms underlying the development of AKI. It is clear, however, that instead of a single mechanism being responsible for its etiology, AKI is associated with an entire orchestra of failing cellular mechanisms [5–8].

It is well known that reactive oxygen species (ROS) are fundamentally implicated as primary culprits in the pathophysiology of renal I/R injury and consequent AKI. The excess generation of ROS and decreases in antioxidant defenses are known to contribute to I/R injury. Superoxide dismutase (SOD), an ubiquitous intrinsic biological antioxidant, catalyzes the dismutation of superoxide anions into oxygen and hydrogen peroxide. Tempol (4-hydroxy-2,2,6,6-tetramethyl piperidinoxyl) is a membrane-permeable, metal-independent SOD mimetic specific for superoxide anions (O_2_^−^). Several studies have demonstrated that tempol may reduce renal I/R injury through its free radical scavenging activity [9, 10].

In a series of recent reviews, we have described that our hypothesis that a disturbed balance between oxygen, nitric oxide (NO), and ROS might form an important component of the pathogenesis of I/R-induced AKI [11, 12, 8]. In the present study, we aimed to test whether the proven protective effects of tempol are indeed associated with improved renal oxygenation and NO levels in a short-term rat model of renal transplantation.

## Methods

### Animals

All experiments in this study were approved by the Institutional Animal Experimentation Committee of the Academic Medical Center of the University of Amsterdam. Care and handling of the animals were in accordance with the guidelines for Institutional and Animal Care and Use Committees. The study has been carried out in accordance with the Declaration of Helsinki. The experiments were performed on 24 Sprague-Dawley rats (Harlan Netherlands BV, Horst, The Netherlands) with a mean ± SD body weight of 348 ± 21 g.

### Surgical preparation

All the animals were anesthetized with an intraperitoneal injection of a mixture of 75 mg/kg ketamine (Nimatek®, Eurovet, Bladel, The Netherlands), 0.5 mg/kg dexmedetomidine (Dexdomitor, Pfizer Animal Health BV, Capelle aan den IJssel, The Netherlands), and 0.05 mg/kg atropine-sulfate (Centrafarm Pharmaceuticals BV, Etten-Leur, The Netherlands). After preparing a tracheotomy, the animals were mechanically ventilated with a FiO_2_ of 0.4. Body temperature was maintained at 37 ± 0.5 °C during the entire experiment by an external thermal heating pad. Ventilator settings were adjusted to maintain end-tidal pCO_2_ between 30 and 35 mmHg and arterial pCO_2_ between 35 and 40 mmHg.

For drug and fluid administration and hemodynamic monitoring, vessels were cannulated with polyethylene catheters with an outer diameter of 0.9 mm (Braun, Melsungen, Germany). A catheter in the right carotid artery was connected to a pressure transducer to monitor mean arterial blood pressure (MAP) and heart rate. The right jugular vein was cannulated for continuous infusion of Ringer’s lactate (Baxter, Utrecht, The Netherlands) at a rate of 15 mL/kg/h and maintenance of anesthesia. The right femoral artery was cannulated for drawing blood samples and the right femoral vein for fluid resuscitation.

The left kidney was exposed, decapsulated, and immobilized in a Lucite kidney cup (K. Effenberger, Pfaffingen, Germany) via ~4 cm incision in the left flank in each animal. The renal vessels were carefully separated under preservation of nerves and the adrenal gland. A perivascular ultrasonic transient time flow probe was placed around the left renal artery (type 0.7 RB Transonic Systems Inc., Ithaca, NY, USA) and connected to a flow meter (T206, Transonic Systems Inc., Ithaca, NY, USA) to continuously measure renal blood flow (RBF). An estimation of the renal vascular resistance (RVR) was made as: RVR (dynes.sec.cm^−5^) = (MAP/RBF) × 80. The left ureter was isolated, ligated, and cannulated with a polyethylene catheter for urine collection.

After the surgical preparation, one optical fiber was placed 1 mm above the decapsulated kidney and another optical fiber was placed 1 mm above the renal vein to measure renal microvascular and venous oxygenation using phosphorimetry (explained in more detail below). A small piece of aluminum foil was placed on the dorsal side of the renal vein to prevent contribution of the underlying tissues to the phosphorescence signal in the venous pO_2_ measurements. Oxyphor G2, a two-layer glutamate dendrimer of tetra-(4-carboxy-phenyl) benzoporphyrin (Oxygen Enterprises Ltd., Philadelphia, PA, USA), was subsequently infused (i.e., 6 mg/kg IV over 5 min), followed by 30 min of stabilization time. The surgical field was covered with a humidified gauze compress throughout the entire experiment to prevent drying of the exposed tissues.

### Experimental protocol

After a stabilization period of 30 min, the animals were randomly divided into four groups of six: (1) no I/R, no tempol (CTRL); (2) no I/R, but with tempol (TMPL); (3) I/R without tempol (I/R); and (4) I/R with tempol (I/R + TMPL). Ischemia/reperfusion was induced by 30-min non-destructive clamping of the renal artery. The tempol-treated animals received 200 μmol/kg/h of 4-hydroxy-TEMPO (tempol) intravenously 15 min prior to initiation of I/R. Measurements were performed up to 90 min post-ischemia, and after the experiments, the kidneys were isolated and renal tissue malondialdehyde (oxidative stress marker) and nitric oxide levels were measured.

### Blood variables

Arterial blood samples (0.5 ml) were taken from the femoral artery at baseline (BSLN) and after 15 and 90 min of reperfusion (R15 and R90, respectively). The blood samples were replaced by the same volume of Ringer’s lactate. The samples were analyzed for blood gas values (ABL505 blood gas analyzer; Radiometer, Copenhagen, Denmark), hemoglobin concentration, and hemoglobin oxygen saturation (OSM3; Radiometer, Copenhagen, Denmark). Additionally, plasma creatinine and sodium concentrations were determined in all the samples.

### Renal microvascular and venous oxygenation

Microvascular oxygen tension in the renal cortex (CμPO_2_), outer medulla (MμPO_2_), and renal venous oxygen tension (P_rv_O_2_) were measured by oxygen-dependent quenching of phosphorescence lifetimes of the systemically infused albumin-targeted (and therefore circulation-confined) phosphorescent dye Oxyphor G2 [13]. Oxyphor G2 has two excitation peaks (*λ*_excitation1_ = 440 nm, *λ*_excitation2_ = 632 nm) and one emission peak (*λ*_emission_ = 800 nm). These optical properties allow (near) simultaneous lifetime measurements in microcirculation of the kidney cortex and the outer medulla due to different optical penetration depths of the excitation light [13]. For the measurement of renal venous PO_2_ (P_rv_O_2_), a mono-wavelength phosphorimeter was used [14]. Oxygen measurements based on phosphorescence lifetime techniques rely on the principle that phosphorescence can be quenched by energy transfer to oxygen resulting in shortening of the phosphorescence lifetime. A linear relationship between reciprocal phosphorescence lifetime and oxygen tension (i.e., the Stern-Volmer relation) allows quantitative measurement of PO_2_ [15].

### Renal oxygen delivery and consumption

Arterial oxygen content (AOC) was calculated by (1.31 × hemoglobin × S_a_O_2_) + (0.003 × P_a_O_2_), where S_a_O_2_ is the arterial oxygen saturation and P_a_O_2_ is the arterial partial pressure of oxygen. Renal venous oxygen content (RVOC) was calculated as (1.31 × hemoglobin × S_rv_O_2_) + (0.003 × P_rv_O_2_), where S_rv_O_2_ is the venous oxygen saturation and P_rv_O_2_ is the renal vein partial pressure of oxygen (measured using phosphorimetry). Renal oxygen delivery was calculated as DO_2_ (mL/min) = RBF × AOC. Renal oxygen consumption was calculated as VO_2_ (mL/min) = RBF × (AOC – RVOC).

### Renal function

For analysis of urine volume, creatinine concentration, and sodium (Na^+^) concentration at the end of the protocol, urine samples from the left ureter were collected for 10 min. Creatinine clearance rate (CCR) per gram of renal tissue was calculated with standard formula: CCR [mL/min] = (*U*_C_ × *V*)/*P*_C_, where *U*_C_ is the urine creatinine concentration, *V* is the urine volume per unit time, and *P*_C_ is the plasma creatinine concentration. Renal sodium reabsorption (*T*_Na+_, [mmol/min]) was calculated as *T*_Na+_ = (*P*_Na+_ × CCR) − (*U*_Na+_ × *V*), where *U*_Na+_ is the urine sodium concentration and *P*_Na+_ is the plasma sodium concentration.

### Renal tissue oxidative stress

Renal tissue malondialdehyde (MDA) levels were determined to assess lipid peroxidation as a measure of renal oxidative stress. All kidneys were homogenized in cold 5-mM sodium phosphate buffer. The homogenates were centrifuged at 12,000*g* for 15 min at 4 °C, and supernatants were used for MDA determination. The level of lipid peroxides was expressed as micromoles of MDA per milligram of protein (Bradford assay).

### Renal tissue NO levels

NO undergoes a series of reactions in biological tissues leading to the accumulation of the final products nitrite and nitrate. Thus, the index of the total NO accumulation is the sum of both nitrite and nitrate levels in the tissue samples. To reduce the nitrate and nitrate pressnet in the tissue samples to NO, the samples were put in the reducing agent vanadium (III) chloride (VCl_3_) in 1 mol/L HCl at 90 °C. The VCl_3_ reagent converts nitrite, nitrate, and S-nitroso compounds to NO gas which is guided towards an NO chemiluminescence signal analyzer (Sievers 280i analyzer, GE Analytical Instruments) allowing the direct detection of NO [16]. Within the reaction vessel, NO reacted with ozone to generate oxygen and excited-state NO species, of which the decay is associated with the emission of weak near-infrared chemiluminescence. This signal is detected by a sensitive photodetector and converted to millivolts (mV). The area under the curve of the detected chemiluminescence (mV∙s) represents the amount of NO-ozone reactions in time and thus the amount of bioavailable NO in the tested samples. The ratio of tissue NO to tissue protein content was used for standardization of the NO measurements.

### Data analysis

Data analysis and presentation were performed using GraphPad Prism (GraphPad Software, San Diego, CA, USA). The values are reported as the mean ± SD. Two-way ANOVA for repeated measurements with a Bonferroni post hoc test were used for comparative analysis between groups. A *p* value of <0.05 was considered statistically significant.

## Results

### Systemic and renal hemodynamics and oxygenation

All systemic and renal hemodynamic and oxygenation variables are presented in Tables 1 and 2. MAP and renal VO_2_ remained stable throughout the entire protocol in all the groups. Tempol administration in the sham-operated animals (i.e., without I/R) did not affect any of the systemic and renal hemodynamic and oxygenation variables. I/R without tempol administration led to a significant decrease in RBF (2.5 ± 0.6 mL/min at R15 and 2.4 ± 0.3 mL/min at R90) and DO_2_ (1.05 ± 0.28 mL O_2_/min at R15 and 0.90 ± 0.22 mL O_2_/min at R90) and a significant increase in RVR (3298 ± 955 dyn·s·cm^−5^ at R15 and 3352 ± 426 dyn·s·cm^−5^ at R90). Tempol administration prior to I/R was able to preserve RBF (4.0 ± 0.9 mL/min at R15 and 4.1 ± 1.6 mL/min at R90), DO_2_ (1.61 ± 0.46 mL O_2_/min at R15 and 1.75 ± 0.70 mL O_2_/min at R90), and RVR (1999 ± 471 dyn·s·cm^−5^ at R15 and 2200 ± 1046 dyn·s·cm^−5^ at R90).

**Table 1:** Mean arterial pressure (MAP), renal blood flow (RBF), renal vascular resistance (RVR), renal oxygen delivery (DO_2_), and renal oxygen consumption (VO_2_) at baseline (Bsln) and after 15 and 90 min of reperfusion (R15 and R90, respectively)

	Bsln	R15	R90
MAP [mmHg]			
CTRL	103 ± 7	103 ± 5	96 ± 6
TMPL	103 ± 8	96 ± 8	93 ± 4
I/R	101 ± 10	96 ± 6	98 ± 6
I/R+TMPL	105 ± 11	95 ± 16	96 ± 16
RBF [mL/min]			
CTRL	4.3 ± 1.3	4.1 ± 1.4	3.8 ± 0.5
TMPL	4.2 ± 0.7	3.8 ± 1.0	3.7 ± 1.3
I/R	4.0 ± 0.6	2.5 ± 0.6 ^**CT**^	2.4 ± 0.3 ^**CT**^
I/R+TMPL	4.4 ± 1.0	4.0 ± 0.9 ^**I**^	4.1 ± 1.6 ^**I**^
RVR [dyn.s.cm^-5^]			
CTRL	2060 ± 583	2143 ± 542	2070 ± 240
TMPL	1989 ± 379	2189 ± 712	2223 ± 733
I/R	2064 ± 414	3298 ± 955 ^**CT**^	3352 ± 426 ^**CT**^
I/R+TMPL	1968 ± 454	1999 ± 471 ^**I**^	2200 ± 1046 ^**I**^
DO_2_ [mL O_2_/min]			
CTRL	1.77 ± 0.53	1.65 ± 0.52	1.52 ± 0.22
TMPL	1.75 ± 0.20	1.54 ± 0.18	1.45 ± 0.21
I/R	1.62 ± 0.33	1.05 ± 0.28 ^**CT**^	0.90 ± 0.22 ^**CT**^
I/R+TMPL	1.88 ± 0.42	1.61 ± 0.46 ^**I**^	1.75 ± 0.70 ^**I**^
VO2 [mL O_2_/min/g]			
CTRL	0.12 ± 0.04	0.11 ± 0.02	0.12 ± 0.02
TMPL	0.13 ± 0.07	0.13 ± 0.03	0.11 ± 0.03
I/R	0.13 ± 0.04	0.10 ± 0.03	0.10 ± 0.03
I/R+TMPL	0.14 ± 0.04	0.13 ± 0.05	0.13 ± 0.04

^C^ *p* < 0.05 vs CTRL, ^T^ *p* < 0.05 vs TMPL, ^I^ *p* < 0.05 vs I/R

**Table 2:** Microvascular oxygen tension in renal cortex (CμpO_2_) and medulla (MμpO_2_) at baseline (Bsln), at the end of 30 min of ischemia (Isch), and after 15 and 90 min of reperfusion (R15 and R90, respectively)

	Bsln	Isch	R15	R90
CμpO_2_				
[mmHg]				
CTRL	76 ± 2	70 ± 4	70 ± 4	62 ± 7
TMPL	76 ± 3	71 ± 8	73 ± 6	58 ± 6
I/R	79 ± 4	11 ± 4 ^**CT**^	59 ± 4	44 ± 11 ^**CT**^
I/R+TMPL	77 ± 6	10 ± 4 ^**CT**^	66 ± 9	57 ± 4 ^**I**^
MμpO_2_				
[mmHg]				
CTRL	61 ± 5	57 ± 6	54 ± 4	51 ± 4
TMPL	57 ± 9	56 ± 10	55 ± 7	50 ± 7
I/R	59 ± 6	7 ± 1 ^**CT**^	50 ± 3 ^**C**^	41 ± 5 ^**CT**^
I/R+TMPL	59 ± 5	7 ± 1 ^**CT**^	59 ± 7 ^**I**^	51 ± 2 ^**I**^

^C^ *p* < 0.05 vs CTRL, ^T^ *p* < 0.05 vs TMPL, ^I^ *p* < 0.05 vs I/R

### Renal microvascular oxygenation

Renal microvascular oxygenation in the cortex and medulla decreased quickly during ischemia but normalized immediately upon reperfusion. However, at R90, microvascular oxygenation was again significantly decreased in the I/R-subjected group that did not receive tempol (44 ± 11 mmHg in the cortex and 41 ± 5 mmHg in the medulla) while this was maintained in the I/R-subjected group that did receive tempol (57 ± 4 mmHg in the cortex and 51 ± 2 mmHg in the medulla).

### Renal oxidative stress and NO levels

**Fig. 1:**
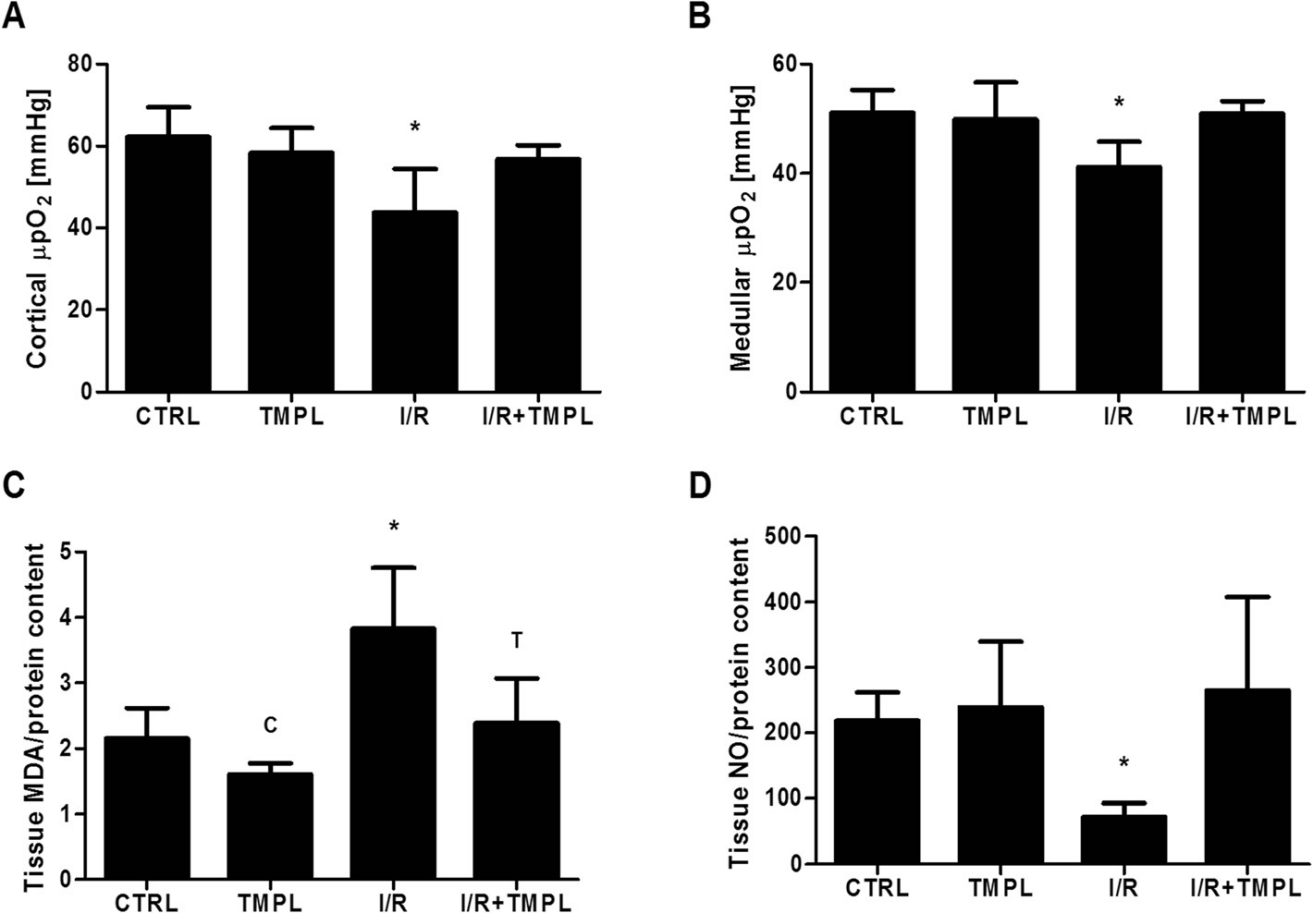
Renal oxygenation, oxidative stress, and nitric oxide (NO) levels at the end of the protocol. **a** Microvascular oxygen tensions (μpO_2_) in the renal cortex; **b** Microvascular oxygen tensions (μpO_2_) in the renal medulla; **c** renal tissue malondialdehyde (MDA) levels normalized to the tissue protein content; and **d** tissue NO levels normalized to the tissue protein content. *p < 0.05 vs all other groups; ^C^ *p* < 0.05 vs the CTRL group; ^T^ *p* < 0.05 vs the TMPL group

The renal microvascular oxygenation, oxidative stress, and NO levels at the end of the protocol are presented in Fig. 1. Tempol administration without I/R injury led to a significant decrease in tissue MDA levels (1.6 ± 0.17) and I/R injury in the absence of tempol led to a significant increase in tissue MDA levels (3.8 ± 0.9). Tempol administration before I/R could partially prevent this increase in MDA levels (2.4 ± 0.7). Tissue NO levels were not affected by tempol administration without I/R injury (240 ± 100), but were significantly decreased after I/R in the absence of tempol (72 ± 21). Tempol administration before I/R could completely normalize the tissue NO levels (265 ± 143). Hence, tempol administration prior to I/R injury reduced renal oxidative stress and normalized renal oxygenation and tissue NO levels.

### Renal function

The renal function variables are presented in Table 3. Tempol administration in the sham-operated animals (i.e., without I/R) did not affect renal function. I/R without tempol administration led to a significant decrease in CCR (0.3 ± 0.1 mL/min at R15) and T_Na+_ (0.04 ± 0.01 mmol/min at R15). Tempol administration prior to I/R could not prevent these reductions in CCR (0.4 ± 0.2 mL/min at R15) and T_Na+_ (0.06 ± 0.03 mmol/min at R15). At R90 these decreases were mostly normalized except for the CCR in the I/R-subjected group that did not receive tempol.

**Table 3:** Creatinine clearance rate (CCR) and sodium reabsoption (T_Na+_) at baseline (Bsln) and after 15 and 90 min of reperfusion (R15 and R90, respectively)

	Bsln	R15	R90
CCR [mL/min]			
CTRL	1.2 ± 0.7	1.3 ± 0.3	1.5 ± 0.7
TMPL	1.1 ± 0.3	1.1 ± 0.3	1.2 ± 0.4
I/R	1.2 ± 0.4	0.3 ± 0.1 ^**CT**^	0.7 ± 0.4 ^**C**^
I/R+TMPL	1.4 ± 0.6	0.4 ± 0.2 ^**CT**^	1.0 ± 0.3
TNa+ [mmol/min]			
CTRL	0.18 ± 0.09	0.18 ± 0.09	0.14 ± 0.07
TMPL	0.15 ± 0.04	0.14 ± 0.04	0.13 ± 0.03
I/R	0.16 ± 0.06	0.04 ± 0.01 ^**CT**^	0.09 ± 0.04
I/R+TMPL	0.20 ± 0.09	0.06 ± 0.03 ^**CT**^	0.14 ± 0.05

^C^ *p* < 0.05 vs CTRL, ^T^ *p* < 0.05 vs TMPL, ^I^ *p* < 0.05 vs I/R

## Discussion

In the present study we aimed to test the hypothesis that scavenging ROS using tempol would not only be associated with reduced renal oxidative stress but also with improved renal oxygenation and NO levels in a short-term rat model of renal I/R. We have found that I/R was associated with a significant increase in tissue MDA levels (marker of oxidative stress) and a significant decrease in tissue NO levels. The decrease in tissue NO was followed by an increase in RVR and consequent decrease in RBF, renal DO_2_, and renal microvascular oxygenation. These disturbances were associated with reduced renal function in terms of sodium reabsorption and creatinine clearance. Pre-ischemic administration of tempol, a known superoxide scavenger, was able to prevent excessive oxidative stress and thereby protect renal tissue NO levels and microvascular oxygenation. Taken together, this led to a preserved renal function after I/R. Furthermore, we have shown that administration of tempol in the absence of I/R leads to a reduction in the renal MDA levels normally present in renal tissue, but did not affect any of the other parameters.

I/R injury is a multi-pathway process in which decreased ROS scavenging and increased ROS generation are particularly important mediators leading to tissue injury [17, 8]. ROS are created in mitochondria [18], and excess ROS injure the mitochondria, impair cellular function, and promote apoptosis [19]. It has previously been shown that antioxidants can decrease cellular and tissue damage by decreasing intracellular ROS levels and suppressing oxidative stress [20–25]. In this study, we showed that tempol reduced lipid peroxidation in renal tissue after renal I/R as reflected by decreased tissue MDA levels [26]. In line, Patel et al. have previously shown that administration of tempone, an unmetabolized form of tempol, reduced I/R-induced injury to peritubular cells by thereby reducing renal dysfunction [20]. They showed, moreover, that this was without the adverse cardiovascular effects observed when using other nitroxyl radical scavenging agents. Noiri et al. also demonstrated that both L-NIL (i.e., a selective inducible nitric oxide synthase (iNOS) inhibitor) and lecithinized SOD administrations improve renal function due to scavenging of peroxynitrite and thereby preventing lipid peroxidation and oxidative damage to DNA [27].

In this study, tempol effectively prevented an I/R-induced decrease in tissue NO concentration. Decreased NO production via endothelial nitric oxide synthase (eNOS) during renal I/R is known to contribute to renal hypoperfusion and renal injury. This has been supported by studies showing that L-arginine (i.e., a precursor of NO) and NO donors improve renal function after I/R [21, 28, 29]. On the other hand, also the administration of iNOS inhibitors has been shown to protect the kidney against I/R injury [30–32, 27]. In the present study, however, the protocol was too short for iNOS expression to occur. Nonetheless, the administration of tempol did scavenge the excess ROS generated during the early phase of I/R and thereby prevented the interaction of eNOS-derived NO and ROS forming peroxynitrite and leaving the NO available for maintenance of microvascular perfusion. Hence, scavenging ROS has a double beneficial effect.

Our study has, however, some limitations. First, this study was performed in rats and the effects of tempol could be different in humans. Second, the duration of renal ischemia was 30 min and measurements were performed up to 90 min post-ischemia and thus long-term effects of I/R and tempol were not studied. Additionally, a longer duration of ischemia might have caused more severe renal dysfunction. Third, we did not measure ROS directly but instead measured MDA as a marker of lipid peroxidation as a result of oxidative stress.

## Conclusions

In conclusion, our study demonstrated that scavenging ROS using tempol not only reduced renal oxidative stress following I/R but also normalized renal tissue NO levels and thereby reduced RVR and improves RBF, renal DO_2_, and renal microvascular oxygenation. Taken together, these effects led to a modest (albeit not statistically significant) improvement of renal function after I/R. This underscores that a disturbed balance between oxygen, NO, and ROS forms an important component of the pathogenesis of I/R-induced AKI and should therefore be taken into account when designing a prevention/treatment strategy for renal I/R injury in transplantation.
